# MicroRNA-221-5p Inhibits Porcine Epidemic Diarrhea Virus Replication by Targeting Genomic Viral RNA and Activating the NF-κB Pathway

**DOI:** 10.3390/ijms19113381

**Published:** 2018-10-29

**Authors:** Hongqing Zheng, Lei Xu, Yuzhong Liu, Cheng Li, Liang Zhang, Tao Wang, Di Zhao, Xingang Xu, Yanming Zhang

**Affiliations:** 1 College of Veterinary Medicine, Northwest A&F University, Yangling 712100, China; zhenghq@nwafu.edu.cn (H.Z.); LiC2018@nwafu.edu.cn (C.L.); eerduosizl@nwafu.edu.cn (L.Z.); wangtao080028@nwsuaf.edu.cn (T.W.); zhaodi1990@nwafu.edu.cn (D.Z.); 2 College of Life Sciences, Northwest A&F University, Yangling 712100, China; xulei@nwafu.edu.cn; 3 LangFangRuiKang Feed Co., Ltd., Guangyang East Road, Economic Development Zone, Langfang 065001, China; tsmm406@gmail.com

**Keywords:** porcine epidemic diarrhea virus (PEDV), microRNA-221-5p, NF-κB, innate immunity

## Abstract

MicroRNAs (miRNAs) are a class of noncoding RNAs involved in posttranscriptional regulation of gene expression and many critical roles in numerous biological processes. Porcine epidemic diarrhea virus (PEDV), the etiological agent of porcine epidemic diarrhea, causes substantial economic loss in the swine industry worldwide. Previous studies reported miRNA involvement in viral infection; however, their role in regulating PEDV infection remains unknown. In this study, we investigated the regulatory relationship between miRNA-221-5p and PEDV infection, finding that miR-221-5p overexpression inhibited PEDV replication in a dose-dependent manner, and that silencing endogenous miR-221-5p enhanced viral replication. Our results showed that miR-221-5p directly targets the 3′ untranslated region (UTR) of PEDV genomic RNA to inhibit PEDV replication, and that miR-221-5p overexpression activates nuclear factor (NF)-κB signaling via p65 nuclear translocation, thereby upregulating interferon (IFN)-β, IFN-stimulated gene 15, and MX1 expression during CH/HBTS/2017 infection. We subsequently identified NF-κB-inhibitor α and suppressor of cytokine signaling 1, negative regulators of the NF-κB pathway, as miR-221-5p targets. These results demonstrated the ability of miR-221-5p to inhibit PEDV replication by targeting the 3’ UTR of the viral genome and activating the NF-κB-signaling pathway. Our findings will aid the development of preventive and therapeutic strategies for PEDV infection.

## 1. Introduction

Porcine epidemic diarrhea (PED), which is caused by infection with PED virus (PEDV), is a highly contagious enteric disease associated with symptoms, including severe enteritis, vomiting, watery diarrhea, and a high mortality rate, in neonatal piglets [1]. The highly pathogenic PEDV variants first appeared in October 2010 in China, during an outbreak characterized by high mortality rates among suckling piglets [2]. PEDV later spread to the United States and other countries, resulting in significant economic losses in the swine industry. PEDV is a single-stranded, positive-sense RNA virus of ~28 kb in length and containing a 5′ cap and a 3′ polyadenylated tail [3]. PEDV belongs to the genus *Alphacoronavirus* of the Coronavirus family, and encodes two polyproteins (pp1a and pp1a/b) that, upon further processing, result in 16 nonstructural proteins, including an accessory protein (ORF3) and four structural proteins: spike (S), membrane (M), envelope (E), and nucleocapsid (N).

MicroRNAs (miRNAs) are small (~23 nt), noncoding RNAs that regulate gene expression posttranscriptionally by degrading mRNAs and/or inhibiting translation [4]. Mammalian miRNAs target mRNAs primarily by pairing with cis-regulatory sites in the 3′ untranslated regions (UTRs) to direct their posttranscriptional repression [5], with an important target motif identified as a ~7-nt site that matches the seed region of the miRNA [6]. By base pairing to mRNAs, miRNAs mediate translational repression or mRNA degradation to control mRNA stability and translation [7]. During host–virus interactions, miRNAs also play important regulatory roles by inhibiting viral replication through the direct targeting of viral genomic RNA [8,9]. Increasing evidence shows that miRNAs regulate innate immune responses in response to viral infection. miR-146 acts as a negative regulatory loop of the nuclear factor (NF)-κB-signaling pathway by targeting tumor necrosis factor (TNF)-receptor-associated factor (TRAF)6 and interleukin (IL)-1-receptor-associated kinase 1 [10]. Additionally, miR-21 is regulated by the signal transducer and an activator of transcription (STAT)3 pathway, and activates the NF-κB pathway by targeting phosphatase and tensin homolog [11]. Moreover, miR-302c represses NF-κB expression by directly targeting the 3′ UTR of NF-κB-inducing kinase (NIK) [12].

However, regulation of PEDV infection by miRNA is poorly understood. Several studies reported interactions between PEDV and the innate immune system. PEDV infection inhibits type I interferon (IFN) production by translating three virus-encoded protein nucleocapsids (N), viral papain-like protease 2, and non-structural protein 1 and 5, which interfere with components associated with the IFN response and downregulate IFN expression [13,14]. However, the mechanism by which cells initiate natural immune responses to PEDV invasion has not been studied.

After viral infection, viral RNA is recognized by pattern recognition receptors (PRRs) in the cytosol, thereby activating the innate immune response to produce type I interferons (IFN-α/β) and establish an antiviral state. Induction of type I IFN converges with the activation of TRAF family member-associated NF-κB-activator-binding kinase 1/IκB kinase (IKK)i, IKKα/β, and mitogen-activated protein kinases which, in turn, promote the phosphorylation and activation of IFN-regulatory factor (IRF)3, NF-κB, and activating protein (AP)-1, respectively. For NF-κB activation, PRRs initiate a signaling cascade resulting in the IKK phosphorylation, which subsequently phosphorylates and ubiquitinates IκB-α bound to NF-κB in resting cells, and results in IκB-α proteasomal degradation, thereby allowing NF-κB nuclear translocation to transcriptionally activate its gene targets [15]. Excessive activation of innate immunity is a hallmark of serious diseases; therefore, organisms have evolved mechanisms to negatively modulate the pathway. IκB negatively regulates the NF-κB pathway by binding to NF-κB, resulting in its sequestration in the cytoplasm. Additionally, suppressor of cytokine signaling 1 (SOCS1) is a negative regulator of Janus kinase (JAK)-STAT signaling [16], and also binds to p65 in the nucleus and acts a ubiquitin ligase to limit prolonged p65-mediated signaling [17].

To identify potent PEDV inhibitors, 30 PEDV strains were used to predict miRNA target sites (TSs), and conservation analysis was performed on genomic RNAs from group 1 and 2 PEDV strains (Table 1). Additionally, we used RegRNA [18] and ViTa [19] to screen potential miRNAs against PEDV infection. Our results revealed a highly conserved miR-221-5p TS in the 3′ UTR of the PEDV genome, with subsequent experiments confirming miR-221-5p suppression of PEDV infection by directly targeting the 3′ UTR of the viral genome. Moreover, we found that miR-221-5p activated the NF-κB pathway by targeting two negative regulators in MARC-145 cells, thereby suggesting a regulatory mechanism wherein host miRNA activates the NF-κB pathway during viral infection.

## 2. Results

### 2.1. miR-221-5p Is Upregulated During PEDV Infection

**Figure 1:**
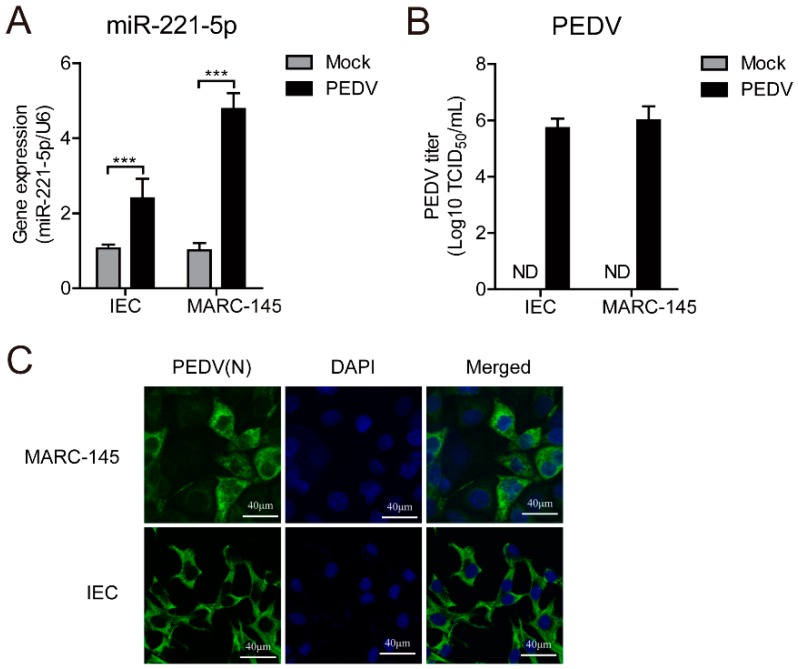
Viral titer of porcine epidemic diarrhea virus (PEDV)-infected cells. (**A**) Real-time qRT-PCR analysis of miR-221-5p expression at 24 h post-infection. Data were normalized to U6 expression. (**B**) Intestinal epithelial cells (IECs) were infected with PEDV CV777, and MARC-145 cells were infected with CH/HBTS/2017 at a multiplicity of infection (MOI) of 1. After 24 h, we determined the tissue culture infectious dose (TCID_50_) value associated with the viral titer. (**C**) Immunofluorescence analysis of intracellular PEDV N protein levels at 24 h post-infection. Cells were fixed and stained with DAPI (blue), with green representing PEDV N protein staining. Scale bar = 40 µm. Comparisons between groups were determined by Student’s *t* test. *** *p* < 0.001.

We predicted miRNAs targeting conserved regions of the viral genome using ViTa [19], identifying miR-221-5p as targeting the 3′ UTR of this region. A previous study confirmed that miR-221-5p expression was induced by viral infection [20]; therefore, we chose this miR for further analysis. To explore whether PEDV infection upregulates miR-221-5p expression, we infected MARC-145 cells with the PEDV strain CH/HBTS/2017 at a multiplicity of infection (MOI) of 1. After 24 h, real-time quantitative reverse transcription polymerase chain reaction (qRT-PCR) results confirmed that miR-221-5p expression was significantly upregulated in PEDV-infected MARC-145 cells (Figure 1A), with this finding verified in PEDV CV777-infected porcine intestinal epithelial cells (IECs) [21]. The 50% tissue culture infectious dose (TCID_50_) and immunofluorescence analysis were performed to confirm virus infection (Figure 1B,C). These results suggested that miR-221-5p expression was activated by PEDV infection.

### 2.2. miR-221-5p Inhibits PEDV Replication in IECs and MARC-145 Cells

**Figure 2:**
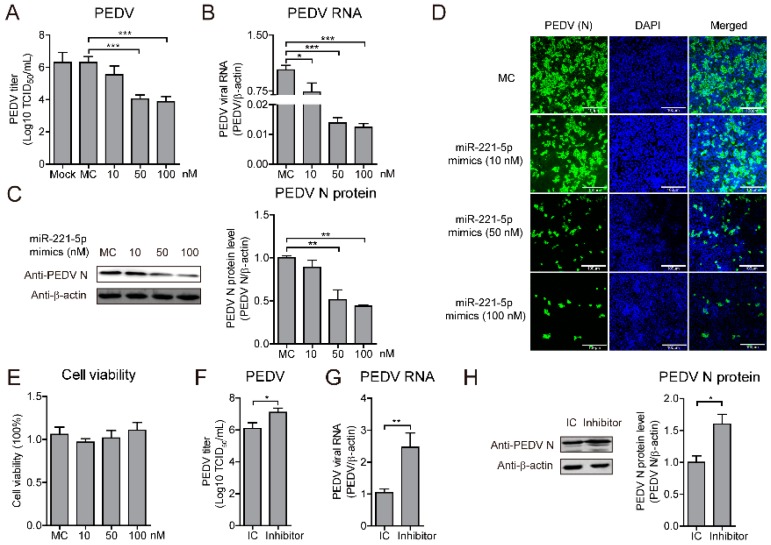
miR-221-5p inhibits PEDV replication in MARC-145 (African green monkey kidney epithelial cells). (**A**–**E**) MARC-145 cells were transfected with miR-221-5p mimics at different concentrations (10, 20, 50, and 100 nM) or mimic control (MC) for 24 h, followed by infection with CH/HBTS/2017 for an additional 24 h. (**A**) TCID_50_ analysis of viral titers. Data represent the mean ± SD of three independent experiments and measured in technical triplicate. (**B**) Real-time qRT-PCR analysis of intracellular virus genomic RNA (normalized to *β-actin* expression). Data represent the mean ± SD of three independent experiments and measured in technical duplicate. (**C**) Immunoblot analysis of intracellular PEDV N protein levels. The intensity represents PEDV N protein level (top) normalized to that of β-actin across three independent experiments. (**D**) Immunofluorescence analysis of intracellular PEDV N protein levels. Scale bar = 100 µm. (**E**) MTT assay to assess cell viability. Data represent the mean ± SD of three independent experiments and measured in technical triplicate. (**F**–**H**) MARC-145 cells were transfected with miR-221-5p inhibitors or IC at 50 nM for 12 h, followed by infection with CH/HBTS/2017 for an additional 36 h. (**F**) TCID_50_ analysis of viral titers. Data represent the mean ± SD of three independent experiments and measured in technical triplicate. (**G**) Real-time qRT-PCR analysis of intracellular viral genomic RNA (normalized to *β-actin* expression). Data represent the mean ± SD of three independent experiments and measured in technical duplicate. (**H**) Immunoblot analysis of intracellular PEDV N protein levels. The intensity represents PEDV N protein level (top) normalized to that of β-actin across three independent experiments. Comparisons between groups were determined by Student’s *t* test. * *p* < 0.05; ** *p* < 0.01; *** *p* < 0.001.

Since PEDV infection upregulates miR-221-5p expression, we evaluated the ability of miR-221-5p to inhibit viral infection. MARC-145 cells were transfected with various concentrations of miR-221-5p mimics, and infected with PEDV at an MOI of 0.1, followed by determination of TCID_50_ value along with real-time qRT-PCR and Western blot. Our results showed that miR-221-5p significantly suppressed PEDV viral load, and that viral RNA and protein were significantly inhibited by miR-221-5p in a dose-dependent manner; however, cell viability was not affected (Figure 2A–D).

**Figure 3:**
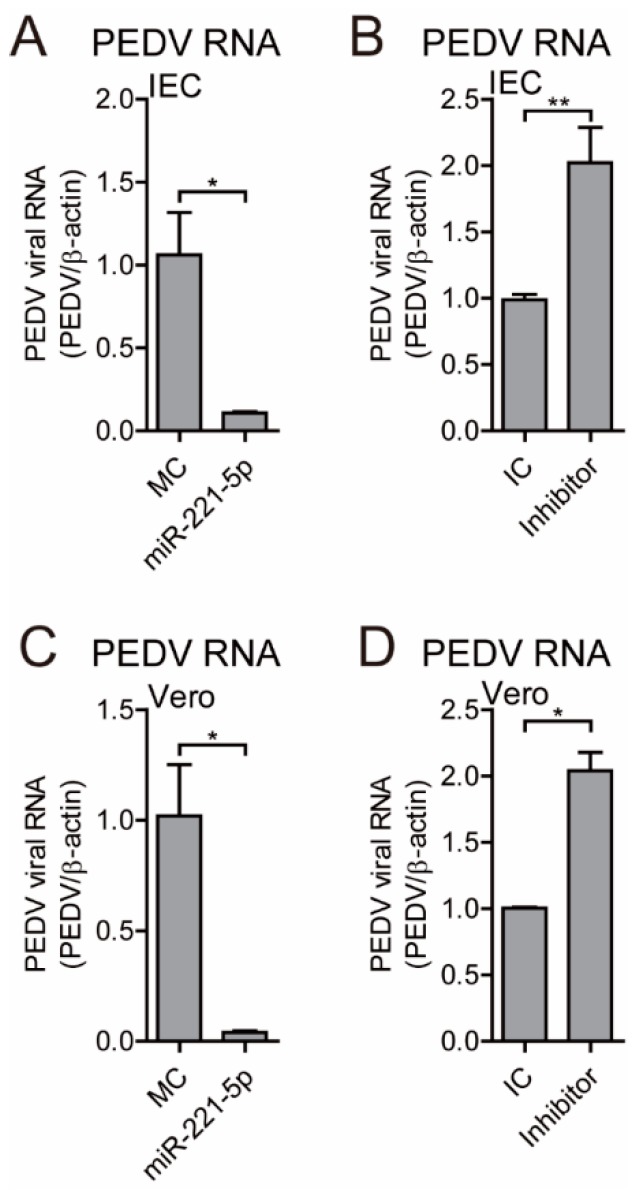
miR-221-5p inhibits PEDV replication in IECs and Vero cells. Real-time qRT-PCR analysis of intracellular viral genomic RNA (normalized to *β-actin* expression). IECs were transfected with miR-221-5p mimics (**A**) or inhibitor (**B**) at 50 nM for 12 h, followed by infection with PEDV CV777 for an additional 36 h. Vero cells were transfected with miR-221-5p mimics (**C**) or inhibitor (**D**) at 50 nM for 12 h, followed by infection with PEDV CH/HBTS/2017 for an additional 36 h. Data represent the mean ± SD of three independent experiments and measured in technical duplicate. Comparisons between groups were determined by Student’s *t* test. * *p* < 0.05; ** *p* < 0.01.

To confirm miR-221-5p-specific inhibition of PEDV replication, MARC-145 cells were transfected with a miR-221-5p inhibitor, which is a chemically modified single-stranded RNA that is complementary to the mature microRNA sequence, or an inhibitor control (IC), and infected with PEDV at an MOI of 0.1. The results showed that the miR-221-5p inhibitor significantly increased PEDV viral titer, as well as viral RNA and protein levels (Figure 2E–H). Additionally, evaluation of the anti-PEDV potential of miR-221-5p in IECs and Vero cells revealed significant inhibition of viral replication, whereas replication was significantly increased in cells transfected with the miR-221-5p inhibitor (Figure 3). These results suggested that miR-221-5p inhibited PEDV replication.

### 2.3. miR-221-5p Suppresses PEDV Infection by Binding to the 3′ UTR of Viral Genome

**Figure 4:**
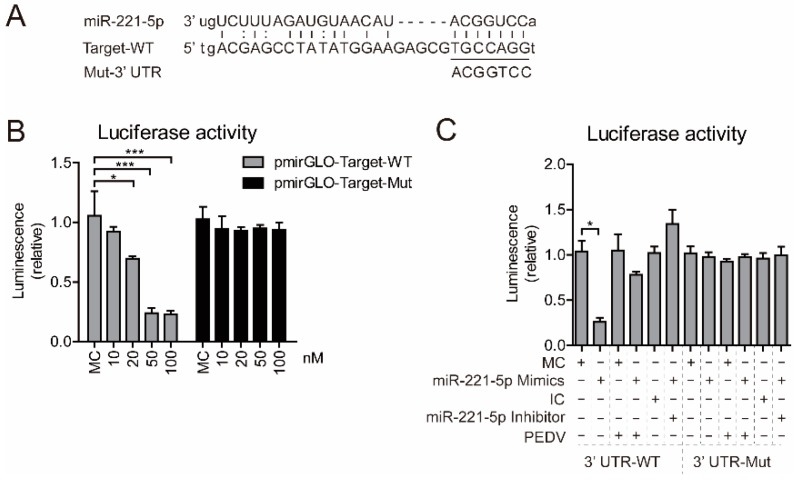
miR-221-5p directly targets the 3′ untranslated region (UTR) of PEDV genomic RNA. (**A**) Diagram of the predicted miR-221-5p-binding site in PEDV genomic RNA. Seed sequences of miR-221-5p are underlined, and mutated sequences are shown. (**B**) Luciferase activity in MARC-145 cells were co-transfected with wild type (WT) (pmirGLO-Target-WT) or mutant (pmirGLO-Target-Mut) luciferase-reporter vector, along with different concentrations of miR-221-5p mimics (10, 20, 50, and 100 nM) for 24 h. (**C**) Luciferase activity in MARC-145 cells in the presence or absence of PEDV CH/HBTS/2017 infection at an MOI of 0.1 for 2 h, followed by co-transfection with miR-221-5p mimics or an miR-221-5p inhibitor. Data in (**B**,**C**) represent the mean ± SD of three independent experiments and measured in technical duplicate. Comparisons between groups were determined by Student’s *t* test. * *p* < 0.05; ** *p* < 0.01; *** *p* < 0.001 (Student’s *t* test). “+” treated; ”−“ untreated.

Cellular miRNAs affect RNA viruses by altering host gene expression [22,23] or by directly binding to viral genomic RNA [24,25,26]. We identified conserved miR-221-5p target sites (TSs) in the 3′ UTR of the viral genomes of 30 PEDV strains (Table 1) [27]. To investigate whether miR-221-5p directly bound the viral genome, we constructed two firefly luciferase reporter-gene plasmids: pmirGLO-target-wild-type (WT) containing the predicted TSs in the 3′ UTR and pmirGLO-target-Mut, where the seed nucleotides of the TSs were mutated (Figure 4A). Upon miR-221-5p binding to the pmirGLO-target-WT construct, the luciferase activity would be downregulated, due to base pairing between the seed region of miR-221-5p and the TS.

Following co-transfection of the pmirGLO-target-WT construct with miR-221-5p mimics into HEK293T cells, we observed that the miR-221-5p mimics significantly inhibited luciferase activity in a dose-dependent manner, with the highest inhibition percentage measured at 90% in the presence of both 50 and 100 nM miR-221-5p mimics (Figure 4B). Notably, the luciferase activity of in cells transfected with the 3′ UTR-Mut remained unchanged, confirming that miR-221-5p specifically binds to the TS. We then investigated whether the downregulated luciferase activity associated with the 3′ UTR-WT could be rescued in the presence of an miR-221-5p inhibitor or by PEDV infection. The results showed significant decreases in the luciferase activity of 3′ UTR-WT in cells transfected with the miR-221-5p mimics (up to 20% relative to controls) (Figure 4C), whereas this decrease was reversed in cells transfected with the miR-221-5p inhibitor or infected with PEDV, suggesting that the miR-221-5p mimics targeted the viral genome rather than the luciferase-reporter sequence. This result demonstrated that PEDV genomic RNA harbors the miR-221-5p TS, which enables miR-221-5p-mediated repression of PEDV infection.

### 2.4. miR-221-5p Increases Type I Interferon Production during PEDV Infection

**Figure 5:**
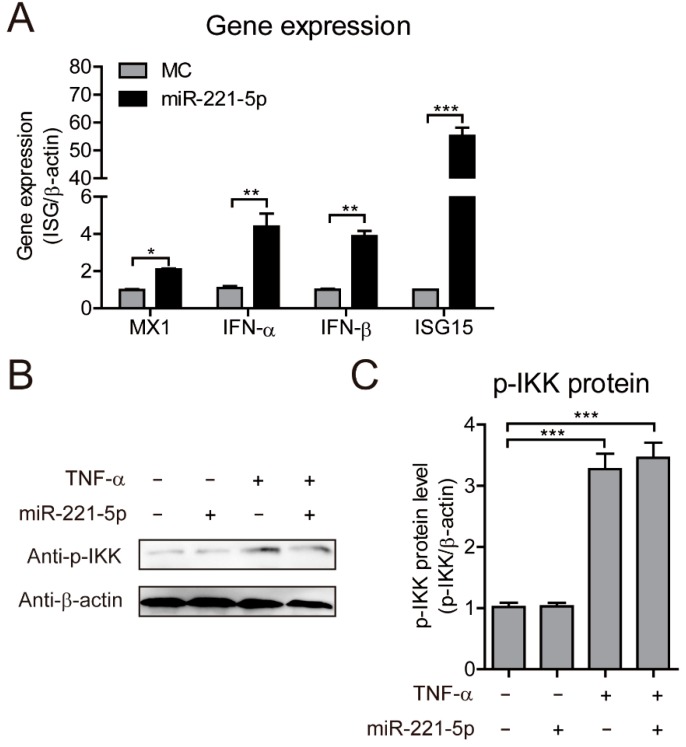
miR-221-5p increases type I IFN and ISG expression during PEDV infection. (**A**) Real-time qRT-PCR analysis of *IFN-α*, *IFN-β*, *MX1*, and *ISG15* mRNA levels (normalized to *β-actin* expression) in MARC-145 cells transfected with 50 nM miR-221-5p mimics or MC for 24 h, followed by infection with CH/HBTS/2017 at an MOI of 0.1 for 24 h. Data represent the mean ± SD of three independent experiments, and measured in technical duplicate. (**B**) Immunoblot analysis of phosphorylated IKK (p-IKK) levels in MARC-145 cells transfected with 50 nM miR-221-5p mimics or MC for 24 h, followed by infection with CH/HBTS/2017 strain at an MOI of 0.1 for 24 h, and stimulation with 15 ng/mL for 12 h. (**C**) The intensity represents p-IKK levels normalized against that for β-actin across three independent experiments. Comparisons between groups were determined by Student’s *t* test. * *p* < 0.05; ** *p* < 0.01; *** *p* < 0.001. “+” treated; ”−“ untreated.

miR-221 increases the antiviral potential of IFN-α [20]. To determine whether miR-221-5p inhibits PEDV replication through activation of the innate immune response, we measured the expression of cytokines, such as IFNs, and IFN-stimulated genes (ISGs), such as *MX1* and *ISG15*. Real-time qRT-PCR analysis following miR-221-5p transfection during PEDV infection revealed that miR-221-5p enhanced cytokine and ISG expression during CH/HBTS/2016 infection (Figure 5A), thereby suggesting a role for miR-221-5p in regulating the innate immune response. To rule out possible miRNA-mediated activation of Toll-like receptor (TLR)3 as a ligand, we determined the phosphorylation level of IKK, a hallmark of TLR3 activation (Figure 5B). The results showed that IKK was not phosphorylated following miR-221-5p transfection, thereby confirming that upregulated IFN and ISG expression was not attributable to TLR3 activation. These results showed that miR-221-5p upregulated IFN and ISG expression to mediate the innate immune response against PEDV infection in MARC-145 cells.

### 2.5. miR-221-5p Activates NF-κB Signaling by Increasing p65 Nuclear Translocation

**Figure 6:**
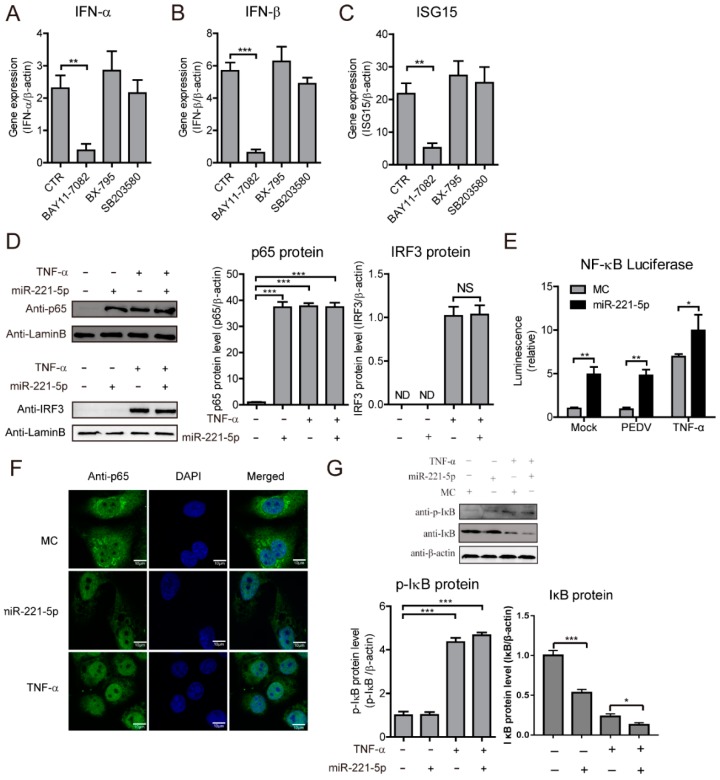
miR-221-5p activates the NF-κB pathway by increasing p65 nuclear translocation. Real-time qRT-PCR analysis of *IFN-α* (**A**), *IFN-β* (**B**), and *ISG15* (**C**) mRNA levels (normalized to *β-actin* expression) in PEDV-infected MARC-145 cells (24 h; CH/HBTS/2017, MOI = 0.1) transfected with 50 nM miR-221-5p mimics or MC for 6 h, followed by treatment with different inhibitors (BAY11-7082, 10 µM; BX-795, 10 µM; or SB203580, 50 µM) for 48 h. Data represent fold changes in the indicated genes relative to MC following miR-221-5p transfection, and are presented as the mean ± SD of three independent experiments and measured in technical duplicate. (**D**) Immunoblot analysis of p65 and IRF3 levels in MARC-145 cells transfected with 50 nM miR-221-5p mimics or MC for 24 h, followed by infection with PEDV CH/HBTS/2017 at an MOI of 0.1 for 24 h. The intensity represents p65 and IRF3 protein levels (right) normalized against that for β-actin across three independent experiments. (**E**) Luciferase activity in MARC-145 cells co-transfected with a dual luciferase-reporter system (PNiFty-luc and pRL-TK, the activities of which indicate NF-κB-promoter activation) and infected with PEDV CH/HBTS/2017 at an MOI of 0.1 for 6 h, or treated with 15 ng/mL TNF-α for 12 h. (**F**) Immunofluorescence analysis of MARC-145 cells transfected with 50 nM miR-221-5p mimics or MC and infected with PEDV CH/HBTS/2017 at an MOI of 0.1 for 24 h. Cells treated with 15 ng/mL TNF-α for 20 min served as the positive controls. Cells were fixed and stained (DAPI, blue; p65 protein, green). Scale bar = 10 µm. (**G**) Immunoblot analysis of p-IκB and IκB levels in MARC-145 cells transfected with 50 nM miR-221-5p mimics or MC for 24 h and infected with PEDV CH/HBTS/2017 at an MOI of 0.1 for 24 h. Intensities representing p-IκB and IκB levels (right) were normalized against that for β-actin across three independent experiments. Comparisons between groups were determined by Student’s *t* test. * *p* < 0.05; ** *p* < 0.01; *** *p* < 0.001; NS *p* > 0.5. “+” treated; ”−“ untreated.

Previous studies reported miR-221 involvement in regulating NF-κB-pathway activation [28,29]. To investigate whether miR-221-5p regulates IFN and ISG expression through the NF-κB pathway, we pretreated PEDV-infected MARC-145 cells with inhibitors of NF-κB (BAY11-7082), IRF3/IRF7 (BX-795), or AP-1 (SB203580), followed by miR-221-5p transfection. We observed that *IFN-α*, *IFN-β*, and *ISG15* expression was induced by miR-221-5p transfection and PEDV infection (Figure 6A–C), and that NF-κB inhibition significantly attenuated these levels, whereas inhibition of AP-1 and IRF3/IRF7 did not, suggesting that miR-221-5p activated the expression of IFNs and ISGs through the NF-κB pathway. To confirm that miR-221-5p activates the NF-κB pathway, we determined p65 and IRF3 protein levels in the nucleus by Western blot, with results showing elevations in nuclear p65 levels following miR-221-5p transfection, although a similar trend was not observed for IRF3 (Figure 6D). We then constructed a luciferase-reporter system (PNiFty-luc) to indicate NF-κB-promoter activation. Our results revealed significant increases in NF-κB-promoter-specific luciferase activity following miR-221-5p transfection of MARC-145 cells (Figure 6E). Additionally, NF-κB-promoter activation induced by PEDV infection and TNF-α were further enhanced in the presence of miR-221-5p.

To explore whether NF-κB-promoter activation was attributed to p65 nuclear translocation, we examined the cellular location of p65 following miR-221-5p transfection using confocal microscopy. We observed a significant increase in p65 nuclear translocation in miR-221-5p-transfected cells, as well as in TNF-α-treated cells, relative to levels observed in controls (Figure 6F), indicating that miR-221-5p promoted p65 nuclear translocation. To determine which protein(s) in in the NF-κB pathway are activated by miR-221-5p, we performed Western blot analysis to detect protein levels of IκB and p-IκB. We found that IκB levels were downregulated, whereas no significant change in p-IκB levels were observed (Figure 6G), suggesting that miR-221-5p might activate the NF-κB pathway through the downregulation of IκB, in order to promote p65 nuclear translocation. These results demonstrated that miR-221-5p activated the expression of IFNs and ISGs via NF-κB-pathway activation through IκB downregulation to promote p65 nuclear translocation.

### 2.6. NFKBIA and SOCS1 Are miR-221-5p Targets

**Figure 7:**
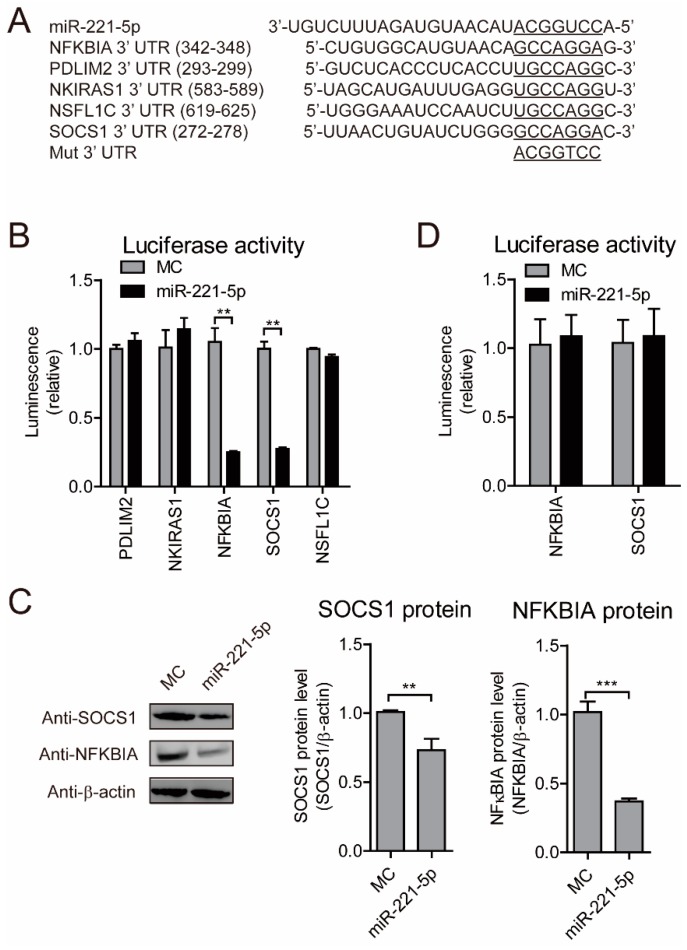
*NFKBIA* and *SOCS1* are miR-221-5p targets. (**A**) Schematic representation of base pairing between miR-221-5p and the 3′ UTRs of *Macaca mulatta NFKBIA* (also known as IκBα), *PDLIM2*, *NKIRAS1*, *NSFL1C*, and *SOCS1*. (**B**) Luciferase activity in 293T cells co-transfected with miR-221-5p and plasmids containing the 3′ UTRs of *NFKBIA*, *PDLIM2*, *NKIRAS1*, *NSFL1C*, and *SOCS1*, respectively, for 48 h. Data were normalized against firefly luciferase activity. (**C**) Immunoblot analysis of NFKBIA and SOCS1 levels in MARC-145 cells transfected with 50 nM miR-221-5p mimics or MC for 24 h, and infected PEDV CH/HBTS/2017 at an MOI of 0.1 for 24 h. Intensity representing NFKBIA and SOCS1 levels (right) normalized against that for β-actin across three independent experiments. (**D**) Luciferase activity in 293T cells co-transfected with miR-221-5p and plasmids containing *NFKBIA* and *SOCS1* 3′ UTRs. Comparisons between groups were determined by Student’s *t* test. ** *p* < 0.01. *** *p* < 0.001.

To identify other miR-221-5p target genes, predictive analysis was performed using TargetScan. The results showed several putative miR-221-5p-binding sites in the 3′ UTRs of *NFKBIA* (also known as IκBα), PDZ- and LIM-domain-containing protein 2 (*PDLIM2*), NF-κB-interacting Ras-like 1 (*NKIRAS1*), *N*-ethylmaleimide-sensitive factor L1 cofactor (*NSFL1C*), and *SOCS1* (Figure 7A). To verify this, six luciferase reporter plasmids, containing the seed sequence of the miR-221-5p-binding site at 3′ UTRs of the reporter genes, were constructed and respectively co-transfected into HEK293T cells, along with miR-221-5p mimics or MC. Compared with MC, miR-221-5p mimics reduced the luciferase activities by ~60% and ~70% in cells transfected with the *NFKBIA* and *SOCS1* 3′ UTRs, respectively (Figure 7B), suggesting these transcripts as potential miR-221-5p targets. Western blot confirmed associated reductions in NFKBIA and SOCS1 proteins (Figure 7C). To confirm these results, two luciferase-reporter plasmids harboring mutated *NFKBIA* and *SOCS1* 3′ UTRs were co-transfected into HEK293T cells along with miR-221-5p mimics or MC. We observed no differences in luciferase activities between cells harboring the MC and the miR-221-5p mimics (Figure 7D), thereby confirming the affinity of miR-221-5p for the 3′ UTRs of *NFKBIA* and *SOCS1*, and suggesting a potential role for miR-221-5p inhibiting PEDV infection through these host genes.

### 2.7. NFKBIA and SOCS1 Suppress IFN-β Expression to Promote PEDV Replication

**Figure 8:**
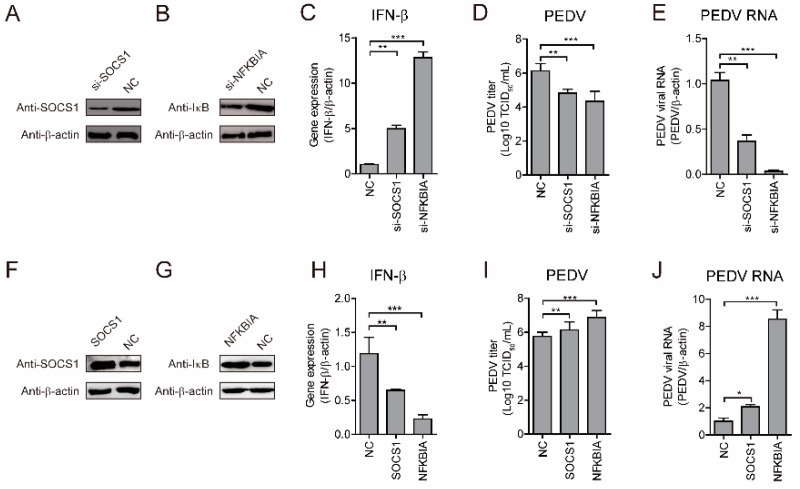
NFKBIA and SOCS1 suppress IFN-β expression to promote PEDV replication. (**A**–**E**) MARC-145 cells were transfected with siRNA control (NC), si-NFKBIA, or si-SOCS1 for 48 h and infected with PEDV CH/HBTS/2017 at an MOI of 0.1 for 24 h. (**A**,**B**) Western blot confirmed the knockdown of SOCS1 and IκB. (**F**–**J**) MARC-145 cells were transfected with Flag control (NC), Flag-NFKBIA, or Flag-SOCS1 for 48 h, and infected with PEDV CH/HBTS/2017 at an MOI of 0.1 for 24 h. (**F**,**G**) confirmed the overexpression of SOCS1 and IκB. (**C**,**H**) Real-time qRT-PCR analysis of *IFN-β* expression (normalized to *β-actin* expression). (**D**,**I**) TCID_50_ analysis of viral titers. (**E**,**J**) Real-time qRT-PCR analysis of intracellular levels of viral genomic RNA (normalized to *β-actin* expression). (**A**–**F**) Data represent the mean ± SD of three independent experiments and measured in technical duplicate. Comparisons between groups were determined by Student’s *t* test. * *p* < 0.05; ** *p* < 0.01; *** *p* < 0.001.

NFKBIA (IκBα) inhibits the NF-κB pathway, and likely sequesters the p65 and p50 complex in the cytoplasm, and SOCS1 negatively regulates the JAK-STAT pathway, with miR-221 enhancing the antiviral potential of IFNs by downregulating SOCS1 levels [16,20]. To verify the roles of NFKBIA and SOCS1 in regulating PEDV replication, we silenced *NFKBIA* and *SOCS1*, and confirmed knockdown of their respective protein levels (Figure 8A,B). We found that small-interfering (si)RNA silencing of *NFKBIA* and *SOCS1* (si-NFKBIA and si-SOCS1) significantly activated *IFN-β* expression, accompanied by decreases in PEDV viral titer and intracellular levels of viral genomic RNA (Figure 8C–E). Conversely, *NFKBIA* and *SOCS1* overexpression (NFKBIA-Flag and SOCS1-Flag) inhibited *IFN-β* expression and increased viral titer and intracellular levels of viral genomic RNA (Figure 8F–J). These results demonstrated that NFKBIA and SOCS1 suppressed *IFN-β* expression to favor PEDV replication, thereby suggesting essential roles in regulating IFN expression and viral replication.

## 3. Discussion

Although miRNAs are reportedly associated with viral infection [30,31], studies have not addressed their roles in PDEV infection. In this study, we demonstrated that miR-221-5p was upregulated by PEDV infection, and inhibited PEDV replication by directly binding to the 3′ UTR of the viral genome. Additionally, we revealed miR-221-5p targeting of two host gene transcripts (*NFKBIA* and *SOCS1*) in order to activate the innate immune response to suppress PEDV replication.

A previous study identified 214 miRNAs differentially expressed in PEDV-infected PK-15 cells [32]. In the present study, we initially evaluated whether miR-221-5p was upregulated by PEDV infection using MARC-145 cells, which are suitable for studying PEDV infection and innate immune modulation [33]. Additionally, we used PEDV strains CH/HBTS/2017 and CV777, as well as porcine IECs harboring epithelial cell markers, and incapable of withstanding the higher trypsin concentrations (>2.5 µg/mL) necessary for evaluating CV777 infection [21]. Infection with both PEDV strains significantly upregulated miR-221-5p expression in the host cells (Figure 1), suggesting its potential role in cellular response to PEDV infection. A recent study revealed miR-221 induction by vesicular stomatitis virus infection in an ELF4-dependent manner [34]; however, its response to PEDV infection required further investigation.

miRNAs modulate RNA-mediated host responses against viral infection in multiple ways. Mature miRNA is part of an active RNA-induced silencing complex capable of directly binding viral genomic RNA to promote its recognition and degradation [35]. Previous studies reported that miR-181 and miR-23 directly target viral genomic RNA to inhibit porcine reproductive respiratory syndrome virus replication [8,36], and miR-32 targets the primate foamy virus genome to suppress viral replication [30]. Additionally, several IFN-induced miRNAs (miR-196, miR-296, miR-351, miR-431, and miR-448) target the hepatitis C virus (HCV) genome to inhibit HCV replication [37]. However, some miRNAs reportedly promote viral replication, including miR-122, which promotes HCV RNA accumulation by binding to HCV genomic RNA to stimulate its translation [38]. In the present study, we showed that miR-221-5p inhibited PEDV replication by targeting highly conserved regions in viral genomic RNA (Figure 4). Our findings that blockage of endogenous miR-221-5p significantly increased PEDV infection (Figure 2 and Figure 3) demonstrated that miR-221-5p inhibited PEDV replication by binding to viral genomic RNA.

miRNAs indirectly modulate host factors to influence the viral lifecycle. Since innate immunity is an essential defense mechanism against viral invasion, it is possible that miRNAs are involved in the IFN response or the NF-κB activation, which represent critical aspects of signaling pathways associated with innate immunity [39,40,41,42]. A previous study showed that miR-26a, miR-34a, miR-145, and let-7b regulate *IFN-β* expression during viral infection via a negative feedback loop [43]. In the present study, we predicted that miR-221-5p target genes were associated with the TLR- and NF-κB-signaling pathways. In human colorectal cancer cells, miR-221 acts in a positive feedback loop to maintain continuous activation of the NF-κB pathway [44]. These findings suggested a role for miR-221-5p in regulating the NF-κB pathway to alter PEDV replication. Our results showed that miR-221-5p upregulated the expression of the IFNs and ISGs through the NF-κB pathway by downregulating IκB levels to promote p65 nuclear translocation. Moreover, we demonstrated that miR-221-5p bound the 3′ UTR of *SOCS1*, an inhibitor of the NF-κB pathway and negative regulator of the JAK-STAT pathway [16,20]. A recent study reported that SOCS1 binds the NF-κB component p65 in the nucleus as a ubiquitin ligase in order to promote p65 degradation, limit prolonged p65-mediated signaling, and terminate the expression of NF-κB-inducible genes [17]. In the present study, we confirmed that *SOCS1* overexpression significantly downregulated IFN-β levels, whereas *SOCS1* silencing markedly upregulated IFN-β expression. These findings suggested that miR-221-5p activated the NF-κB pathway to suppress PEDV replication.

In conclusion, we demonstrated miR-221-5p upregulation during PEDV infection and revealed its roles in suppressing PEDV replication by directly binding to the 3′ UTR of the viral genome. Additionally, our results revealed that miR-221-5p activated the NF-κB pathway by targeting *NFKBIA* and *SOCS1*, inhibitors of NF-κB signaling and mediators of IFN and ISG production, to inhibit viral infection.

## 4. Materials and Methods

### 4.1. Cells, Viruses, Reagents, and Antibodies

African green monkey kidney cells MARC-145 and Vero E6 were cultivated in Dulbecco’s modified Eagle medium (DMEM; Hyclone, Logan, UT, USA) supplemented with 10% heat-inactivated fetal bovine serum (FBS; PAN-Biotech, Aidenbach, Germany) in a humidified atmosphere of 5% CO_2_ at 37 °C. Vero E6 cells were used for PEDV propagation. IECs were established in our laboratory and used to analyze PEDV CV777 infection [45,46]. PEDV CV777 (NCBI GenBank accession No. KT323979.1) was stored in our laboratory, and IECs were isolated from the ileum of a newborn piglet [47] and cultured in DMEM-F12 Ham’s medium supplemented with 5% FBS. PEDV CH/HBTS/2017 (NCBI GenBank accession No. MH581489) was isolated from an intestinal sample from a piglet infected in 2017. Viral stocks were harvested from infected Vero cell supernatant after observation of an 80% cytopathic effect and stored at −80°C until further use. Viral titer was determined by standard TCID_50_ assay using MARC-145 cells. TNFα was purchased from Sigma-Aldrich (St. Louis, MO, USA), and dissolved in water to a concentration of 100 μg/mL. The inhibitors (AP-1, SB203580; NF-κB, BAY11-7082; and IRF3/7, BX-795) were purchased from Enzo Life Sciences (Farmingdale, NY, USA). The dual luciferase-reporter assay system was purchased from Promega (Madison, WI, USA). Rabbit anti-p-65 (#6856T), rabbit anti-p-IKKα/β (#2697T), rabbit anti-IκBα (#4814T), rabbit anti-IRF3 (#29047), rabbit anti-SOCS1 (#3950T), and anti-β-actin (#4970T) monoclonal antibodies were purchased from Cell Signaling Technology (Danvers, MA, USA). A mouse anti-PEDV N monoclonal antibody was a gift from Prof. Guangzhi Tong [48]. Hoechst 33251 was purchased from Solarbio (Beijing, China). Lipofectamine 2000 transfection reagent was purchased from Invitrogen (Carlsbad, CA, USA). Secondary antibodies for Western blot were purchased from Bioss (Beijing, China), and the Pierce enhanced chemiluminescence (ECL) Western blot substrate was purchased from Zata Pharmaceuticals (Worcester, MA, USA).

### 4.2. Plasmid Construction

Flag-NFKBIA was synthesized by Genecreate (Wuhan, China) and cloned into pCDNA3.1+ using the *Bam*HI and *Xho*I restriction sites. Similarly, Flag-SOCS1 was cloned into pCDNA3.1+ using the *Bam*HI and *Eco*RI restriction sites. Plasmids harboring the 3′ UTRs (pmirGLO-SOCS1, pmirGLO-NFKBIA, pmirGLO-NSFL1C, pmirGLO-PDLIM2, and pmirGLO-NKIRAS) were cloned from the mRNA of *M. mulatta* by RT-PCR, and ligated into the pmirGLO plasmid (Promega, Madison, WI, USA) using primers listed in Table 2. To obtain mutated miR-221-5p TSs within the 3′ UTRs, seed regions were mutated according to previously described mutagenesis protocol [49].

### 4.3. Transfection of miRNA Mimics

All miRNA mimics, inhibitors, and siRNAs listed in Table 2 were synthesized by RiboBio (Guangzhou, China). IECs or MARC-145 cells were seeded in 24-well plates for 12 h, followed by transfection of miRNA mimics (50 nM) or inhibitors (100 nM) for 24 h using Lipofectamine 2000 (Invitrogen), followed by infection with PEDV at an MOI of 0.1. After 36 h or 48 h of incubation, the supernatant was collected to determine viral titer, and cells were fixed for indirect immunofluorescent staining or collected for RNA quantification or Western blot analysis.

### 4.4. Immunofluorescence Assays

Infected MARC-145 samples were fixed with a mixture of cold methanol and acetone (4:1) for 20 min and blocked with 1% bovine serum albumin for 1 h. The sample was then permeabilized with Triton X-100 for 10 min and incubated for 2 h with the PEDV anti-N monoclonal antibody to detect PEDV. The cells were washed three times for 5 min with phosphate-buffered saline (PBS) and incubated with the secondary antibody (Sungene Biotech, Tianjin, China) for 1 h at room temperature. Cell nuclei were counterstained with Hoechst 33251 for 8 min. After three rinses with PBS, transfected cells were visually examined using a laser scanning confocal microscope.

### 4.5. Western Blot

Cells were lysed with radioimmunoprecipitation assay buffer for 30 min on ice, followed by centrifugation at 12,000× *g* for 15 min at 4 °C. Protein samples were added to 5× loading buffer, boiled for 10 min, subjected to sodium dodecyl sulfate polyacrylamide gel electrophoresis, and transferred to polyvinylidene fluoride membranes, which were blocked with 5% nonfat milk in PBS containing Tween-20 (0.02%), and probed using the primary monoclonal antibody. Horseradish peroxidase-conjugated secondary antibodies were used to detect the primary antibodies, and proteins were visualized by ECL.

### 4.6. qRT-PCR Analysis

Total RNA was extracted with RNAiso Plus reagent (TAKARA, Dalian, China) according to manufacturer instructions. Genomic DNA was removed using gDNA Eraser from the reverse transcription kit (TAKARA, Dalian, China), and 1 μg RNA was used for reverse transcription. Quantitative RT-PCR was performed using TB Green PremixExTaq II (TAKARA, Dalian, China). To determine the relative expression of target genes, we used a commercial miRNA first-strand cDNA synthesis kit (TAKARA, Dalian, China) and an miR QPCR kit (TAKARA, Dalian, China) to measure miR-221-5p levels, which were normalized against that of the U6 gene. For coding genes, relative fold changes in expression were normalized against *β-actin* using the 2^−ΔΔ*C*t^ threshold method.

### 4.7. Dual Luciferase-Reporter Assays

The pmirGLO luciferase reporter vector (Promega, Madison, WI, USA) was used for the luciferase assays analyzing potential miR-221-5p TSs in the 3′ UTRs. The target region of the PEDV genome and mRNAs were subcloned into the multiple cloning site of the pmirGLO vector using primers listed in Table 2. PNiFty-luc, a reporter plasmid containing five NF-κB sites, was used to monitor NF-κB activity. Luciferase assays were performed using a dual luciferase-reporter assay system kit (Promega, Madison, WI, USA) according to manufacturer protocol. To determine miR-221-5p targets, HEK293T cells were transfected with pmirGLO plasmids containing cloned 3′ UTR sequences. To detect NF-κB-promoter activity, MARC-145 cells were co-transfected with pNiFty-luc and pRL-TK at a 10:1 ratio. Collected cells were rinsed with cold PBS and lysed with 100 μL 1× passive lysis buffer for 30 min at 37 °C, followed by centrifugation at 12,000× *g* for 30 s. Luciferase values were determined using a Modulus single-tube multimode reader (Spark; Tecan Life Sciences, Männedorf, Switzerland). Values were normalized using *Renilla* luciferase activity as an internal control, and presented in fold-changes. Three independent assays were performed with each assay in triplicate.

### 4.8. Statistical Analysis

Student’s *t* test was used for all statistical analyses. All data are presented as the mean ± SD of three independent experiments. Asterisks indicate statistical significance. * *p* < 0.05. ** *p* < 0.01 and *** *p* < 0.001.

## Figures and Tables

**Table 1:** Prediction of miRNA target sites (TSs) on genomic RNA from group 1 and 2 PEDV strains.

miRNA	PEDV Strains	Score	Energy (kcal/mol)	Strand	miRNA Length (bp)	Gene Length (bp)	Target Start
ssc-miR-221-5p	AH-2012(KC210145.1)	151	−21.53	4497	26	28,039	27,953
ssc-miR-221-5p	CHFJZZ-2012(KC140102.1)	151	−21.53	4500	26	28,038	27,952
ssc-miR-221-5p	CH-GDGZ-2012(KF384500.1)	151	−21.53	4501	26	28,035	27,949
ssc-miR-221-5p	CH-GDZHDM-1401(KX016034.1)	151	−21.53	4488	26	27,993	27,927
ssc-miR-221-5p	CH-GX-2015-750A(KY793536.1)	151	−21.53	4502	26	28,038	27,952
ssc-miR-221-5p	CH-HNAY-2015(KR809885.1)	151	−21.53	4503	26	28,038	27,952
ssc-miR-221-5p	CH-HNQX-3-14(KR095279.1)	151	−21.53	4522	26	27,997	27,911
ssc-miR-221-5p	CH-HNYF-14(KP890336.1)	151	−21.53	4504	26	27,996	27,910
ssc-miR-221-5p	CH-HNZZ47-2016(KX981440.1)	151	−21.53	4505	26	28,124	27,937
ssc-miR-221-5p	CH_hubei_2016(KY928065.1)	151	−21.53	4499	26	28,063	27,948
ssc-miR-221-5p	CH-JLDH-2016(MF346935.1)	151	−21.53	4485	26	28,035	27,949
ssc-miR-221-5p	CH-JX-1-2013(KF760557.2)	151	−21.53	4506	26	28,052	27,952
ssc-miR-221-5p	CH-SCCD-2014(KU975389.1)	151	−21.53	4507	26	28,044	27,952
ssc-miR-221-5p	CH-SD01(KU380331.1)	151	−21.53	4508	26	28,035	27,949
ssc-miR-221-5p	CH-SXYL-2016(MF462814.1)	151	−21.53	4486	26	28,076	27,956
ssc-miR-221-5p	CH-YNKM-8-2013(KF761675.1)	151	−21.53	4519	26	28,038	27,952
ssc-miR-221-5p	CH-ZJCX-1-2012(KF840537.1)	151	−21.53	4520	26	27,992	27,921
ssc-miR-221-5p	CH-ZMDZY-11(KC196276.1)	151	−21.53	4521	26	28,038	27,952
ssc-miR-221-5p	CV777(AF353511.1)	151	−21.53	4496	26	28,033	27,947
ssc-miR-221-5p	DR13(JQ023162.1)	151	−21.53	4510	26	27,931	27,846
ssc-miR-221-5p	GD-1(JX647847.1)	151	−21.53	4511	26	28,047	27,949
ssc-miR-221-5p	GD-B(JX088695.1)	151	−21.53	4512	26	28,038	27,952
ssc-miR-221-5p	HBTS2017(MH581489)	151	−21.53	4489	26	28,066	27,949
ssc-miR-221-5p	JS-HZ2012(KC210147.1)	151	−21.53	4513	26	28,037	27,951
ssc-miR-221-5p	OH1414(KJ408801.1)	151	−21.53	4495	26	28,038	27,952
ssc-miR-221-5p	ON-018(KM189367.2)	151	−21.53	4494	26	28,061	27,952
ssc-miR-221-5p	PC177(KR078300.1)	151	−21.53	4493	26	28,028	27,941
ssc-miR-221-5p	PEDV-SX-2017(KY420075.1)	151	−21.53	4490	26	27,967	27,881
ssc-miR-221-5p	PEDV-SX(KY420075.1)	151	−21.53	4487	26	27,967	27,881
ssc-miR-221-5p	YC2014(KU252649.1)	151	−21.53	4518	26	28,077	27,973
ssc-miR-221-5p	ZL29(KU847996.1)	151	−21.53	4492	26	27,989	27,908

**Table 2:** Sequences of primer pairs used for qRT-PCR and plasmid construction.

Genes	Forward Primer (5′–3′) *	Reverse Primer (5′–3′) *	Ref.	Use
*miR-221-5p-F*	GACCTGGCATACAATGTAGATTTCTGT			RT-qPCR detection of miR-221-5p
*U6*	CTCGCTTCGGCAGCACA	AACGCTTCACGAATTTGCGT		RT-qPCR detection of U6
*miR-221-5p mimics*	ACCUGGCAUACAAUGUAGAUUUCUGU	ACAGAAAUCUACAUUGUAUGCCAGGU		Overexpression of miR-221-5p
*miR-221-5p inhibitor*	ACAGAAAUCUACAUUGUAUGCCAGGU			Silencing of miR-221-5p
*PEDV viral gRNA*	AGTACGGGGCTCTAGTGCAG	GCTTATCCAAATTCTTCAGGCG	[20]	RT-qPCR detection of PEDV viral gRNA
*β-actin*	ATCGTGCGTGACATTAAG	ATTGCCAATGGTGATGAC	[34]	RT-qPCR detection of β-actin
*IFN-α*	CTGCTGCCTGGAATGAGAGCC	CTGCTGCCTGGAATGAGAGCC	[34]	RT-qPCR detection of IFN-α
*ISG15*	CACCGTGTTCATGAATCTGC	CTTTATTTCCGGCCCTTGAT	[34]	RT-qPCR detection of ISG15
*IFN-β*	GATTTATCTAGCACTGGCTGG	CTTCAGGTAATGCAGAATCC	[34]	RT-qPCR detection of IFN-β
*MX1*	TCAAGACACTCATCAGGAAG	CACCACATCCACAACCTT	[34]	RT-qPCR detection of MX1
*pmirGLO-NFκBIA*	GCCGAGCTCGGACCACATTTTATATTTATTG	GCCTCTAGACAGAAGGGTAACACAAAC		To generate pmirGLO-NFκBIA
*pmirGLO-PDLIM2*	GCCGAGCTCGAGTCTCACCCTCACCTTG	GCCTCTAGATCTCCTTCCCACTTC		To generate pmirGLO-PDLIM2
*pmirGLO-NKIRAS1*	GCCGAGCTCCACTTTGGGGTAGTAAGCTA	GCCCTCGAGTAGAGCCCAGTATTTTG		To generate pmirGLO-NKIRAS1
*pmirGLO-NSFL1C*	GCCGAGCTCGCTACATGCACACAGTG	GCCTCTAGAGGAGGTGAGCAGTCTG		To generate pmirGLO-NSFL1C
*pmirGLO-SOCS1*	CGGGAGCTCACCTCTTGAGGGGGTTC	GCCTCTAGAAGGATTCTGCACAGCAG		To generate pmirGLO-SOCS1
*pmirGLO-3′ UTR*	CGGTCTAGAGCTATGGCTTTGCCCTCTAA	CGGGAGCTCCAACACCGTCAGGTCTTCAGT		To generate pmirGLO-3′ UTR

* Underlined sequences indicate restriction enzyme sites added for cloning.

## References

[1] Vlasova A.N., Marthaler D., Wang Q., Culhane M.R., Rossow K.D., Rovira A., Collins J., Saif L.J. (2014). Distinct characteristics and complex evolution of PEDV strains, North America, May 2013–February 2014. Emerg. Infect. Dis..

[2] Sun R.-Q., Cai R.-J., Chen Y.-Q., Liang P.-S., Chen D.-K., Song C.-X. (2012). Outbreak of porcine epidemic diarrhea in suckling piglets, China. Emerg. Infect. Dis..

[3] Egberink H.F., Ederveen J., Callebaut P., Horzinek M.C. (1988). Characterization of the structural proteins of porcine epizootic diarrhea virus, strain CV777. Am. J. Vet. Res..

[4] Bartel D.P. (2009). MicroRNAs: Target recognition and regulatory functions. Cell.

[5] Selbach M., Schwanhausser B., Thierfelder N., Fang Z., Khanin R., Rajewsky N. (2008). Widespread changes in protein synthesis induced by microRNAs. Nature.

[6] Grimson A., Farh K.K., Johnston W.K., Garrett-Engele P., Lim L.P., Bartel D.P. (2007). MicroRNA targeting specificity in mammals: Determinants beyond seed pairing. Mol. Cell.

[7] Filipowicz W., Bhattacharyya S.N., Sonenberg N. (2008). Mechanisms of post-transcriptional regulation by microRNAs: Are the answers in sight?. Nat. Rev. Genet..

[8] Guo X.K., Zhang Q., Gao L., Li N., Chen X.X., Feng W.H. (2013). Increasing Expression of MicroRNA 181 Inhibits Porcine Reproductive and Respiratory Syndrome Virus Replication and Has Implications for Controlling Virus Infection. J. Virol..

[9] Zheng Z., Ke X., Wang M., He S., Li Q., Zheng C., Zhang Z., Liu Y., Wang H. (2013). Human microRNA hsa-miR-296-5p suppresses enterovirus 71 replication by targeting the viral genome. J. Virol..

[10] Taganov K.D., Boldin M.P., Chang K.J., Baltimore D. (2006). NF-kappaB-dependent induction of microRNA miR-146, an inhibitor targeted to signaling proteins of innate immune responses. Proc. Natl. Acad. Sci. USA.

[11] Iliopoulos D., Jaeger S.A., Hirsch H.A., Bulyk M.L., Struhl K. (2010). STAT3 activation of miR-21 and miR-181b-1 via PTEN and CYLD are part of the epigenetic switch linking inflammation to cancer. Mol. Cell.

[12] Gui S., Chen X., Zhang M., Zhao F., Wan Y., Wang L., Xu G., Zhou L., Yue X., Zhu Y. (2015). Mir-302c mediates influenza A virus-induced IFNbeta expression by targeting NF-kappaB inducing kinase. FEBS Lett..

[13] Xing Y., Chen J., Tu J., Zhang B., Chen X., Shi H., Baker S.C., Feng L., Chen Z. (2013). The papain-like protease of porcine epidemic diarrhea virus negatively regulates type I interferon pathway by acting as a viral deubiquitinase. J. Gen. Virol..

[14] Zhang Q., Ma J., Yoo D. (2017). Inhibition of NF-kappaB activity by the porcine epidemic diarrhea virus nonstructural protein 1 for innate immune evasion. Virology.

[15] Kawai T., Akira S. (2011). Toll-like receptors and their crosstalk with other innate receptors in infection and immunity. Immunity.

[16] Yoshikawa H., Matsubara K., Qian G.S., Jackson P., Groopman J.D., Manning J.E., Harris C.C., Herman J.G. (2001). SOCS-1, a negative regulator of the JAK/STAT pathway, is silenced by methylation in human hepatocellular carcinoma and shows growth-suppression activity. Nat. Genet..

[17] Strebovsky J., Walker P., Lang R., Dalpke A.H. (2011). Suppressor of cytokine signaling 1 (SOCS1) limits NFkappaB signaling by decreasing p65 stability within the cell nucleus. FASEB J..

[18] Huang H.Y., Chien C.H., Jen K.H., Huang H.D. (2006). RegRNA: An integrated web server for identifying regulatory RNA motifs and elements. Nucleic Acids Res..

[19] Hsu P.W., Lin L.Z., Hsu S.D., Hsu J.B., Huang H.D. (2007). ViTa: Prediction of host microRNAs targets on viruses. Nucleic Acids Res..

[20] Xu G., Yang F., Ding C.L., Wang J., Zhao P., Wang W., Ren H. (2014). MiR-221 accentuates IFNs anti-HCV effect by downregulating SOCS1 and SOCS3. Virology.

[21] Wang J., Hu G., Gao W., Xu L., Ning P., Zhang Y. (2014). Immortalized porcine intestinal epithelial cell cultures susceptible to porcine rotavirus infection. J. Virol. Methods.

[22] Saxena T., Tandon B., Sharma S., Chameettachal S., Ray P., Ray A.R., Kulshreshtha R. (2013). Combined miRNA and mRNA signature identifies key molecular players and pathways involved in chikungunya virus infection in human cells. PLoS ONE.

[23] Li Z., Cui X., Li F., Li P., Ni M., Wang S., Bo X. (2013). Exploring the role of human miRNAs in virus-host interactions using systematic overlap analysis. Bioinformatics.

[24] Sedano C.D., Sarnow P. (2014). Hepatitis C virus subverts liver-specific miR-122 to protect the viral genome from exoribonuclease Xrn2. Cell Host Microbe.

[25] Ahluwalia J.K., Khan S.Z., Soni K., Rawat P., Gupta A., Hariharan M., Scaria V., Lalwani M., Pillai B., Mitra D. (2008). Human cellular microRNA hsa-miR-29a interferes with viral nef protein expression and HIV-1 replication. Retrovirology.

[26] Trobaugh D.W., Gardner C.L., Sun C., Haddow A.D., Wang E., Chapnik E., Mildner A., Weaver S.C., Ryman K.D., Klimstra W.B. (2014). RNA viruses can hijack vertebrate microRNAs to suppress innate immunity. Nature.

[27] Yang D.Q., Ge F.F., Ju H.B., Wang J., Liu J., Ning K., Liu P.H., Zhou J.P., Sun Q.Y. (2014). Whole-genome analysis of porcine epidemic diarrhea virus (PEDV) from eastern China. Arch. Virol..

[28] Zhao D., Zhuang N., Ding Y., Kang Y., Shi L. (2016). MiR-221 activates the NF-kappaB pathway by targeting A20. Biochem. Biophys. Res. Commun..

[29] Liu Z., Wang C., Jiao X., Zhao S., Liu X., Wang Y., Zhang J. (2016). miR-221 promotes growth and invasion of hepatocellular carcinoma cells by constitutive activation of NFkappaB. Am. J. Transl. Res..

[30] Lecellier C.H., Dunoyer P., Arar K., Lehmann-Che J., Eyquem S., Himber C., Saib A., Voinnet O. (2005). A cellular microRNA mediates antiviral defense in human cells. Science.

[31] Sullivan C.S., Ganem D. (2005). MicroRNAs and viral infection. Mol. Cell.

[32] Huang J., Lang Q., Li X., Xu Z., Zhu L., Zhou Y. (2016). MicroRNA Expression Profiles of Porcine Kidney 15 Cell Line Infected with Porcine Epidemic Diahorrea Virus. Bing Du Xue Bao = Chin. J. Virol..

[33] Zhang Q., Shi K., Yoo D. (2016). Suppression of type I interferon production by porcine epidemic diarrhea virus and degradation of CREB-binding protein by nsp1. Virology.

[34] Du H., Cui S., Li Y., Yang G., Wang P., Fikrig E., You F. (2018). MiR-221 negatively regulates innate anti-viral response. PLoS ONE.

[35] Ingle H., Kumar S., Raut A.A., Mishra A., Kulkarni D.D., Kameyama T., Takaoka A., Akira S., Kumar H. (2015). The microRNA miR-485 targets host and influenza virus transcripts to regulate antiviral immunity and restrict viral replication. Sci. Signal..

[36] Zhang Q., Guo X.K., Gao L., Huang C., Li N., Jia X., Liu W., Feng W.H. (2014). MicroRNA-23 inhibits PRRSV replication by directly targeting PRRSV RNA and possibly by upregulating type I interferons. Virology.

[37] Pedersen I.M., Cheng G., Wieland S., Volinia S., Croce C.M., Chisari F.V., David M. (2007). Interferon modulation of cellular microRNAs as an antiviral mechanism. Nature.

[38] Henke J.I., Goergen D., Zheng J., Song Y., Schuttler C.G., Fehr C., Junemann C., Niepmann M. (2008). microRNA-122 stimulates translation of hepatitis C virus RNA. EMBO J..

[39] Iwasaki A., Pillai P.S. (2014). Innate immunity to influenza virus infection. Nat. Rev. Immunol..

[40] Loving C.L., Osorio F.A., Murtaugh M.P., Zuckermann F.A. (2015). Innate and adaptive immunity against porcine reproductive and respiratory syndrome virus. Vet. Immunol. Immunopathol..

[41] Heim M.H., Thimme R. (2014). Innate and adaptive immune responses in HCV infections. J. Hepatol..

[42] Kawai T., Akira S. (2006). Innate immune recognition of viral infection. Nat. Immunol..

[43] Witwer K.W., Sisk J.M., Gama L., Clements J.E. (2010). MicroRNA regulation of IFN-beta protein expression: Rapid and sensitive modulation of the innate immune response. J. Immunol..

[44] Liu S., Sun X., Wang M., Hou Y., Zhan Y., Jiang Y., Liu Z., Cao X., Chen P., Liu Z. (2014). A microRNA 221- and 222-mediated feedback loop maintains constitutive activation of NFkappaB and STAT3 in colorectal cancer cells. Gastroenterology.

[45] Cao L., Ge X., Gao Y., Ren Y., Ren X., Li G. (2015). Porcine epidemic diarrhea virus infection induces NF-κB activation through the TLR2, TLR3 and TLR9 pathways in porcine intestinal epithelial cells. J. Gen. Virol..

[46] Wang D., Fang L., Shi Y., Zhang H., Gao L., Peng G., Chen H., Li K., Xiao S. (2016). Porcine epidemic diarrhea virus 3C-like protease regulates its interferon antagonism by cleaving NEMO. J. Virol..

[47] Wang J., Hu G., Lin Z., He L., Xu L., Zhang Y. (2014). Characteristic and functional analysis of a newly established porcine small intestinal epithelial cell line. PLoS ONE.

[48] Pan X., Kong N., Shan T., Zheng H., Tong W., Yang S., Li G., Zhou E., Tong G. (2015). Monoclonal antibody to N protein of porcine epidemic diarrhea virus. Monoclon. Antibodies Immunodiagn. Immunother..

[49] Heckman K.L., Pease L.R. (2007). Gene splicing and mutagenesis by PCR-driven overlap extension. Nat. Protoc..

